# Effectiveness of Surgical Deroofing and Carbon Dioxide Laser in Moderate-to-Severe Hidradenitis Suppurativa Patients

**DOI:** 10.7759/cureus.56959

**Published:** 2024-03-26

**Authors:** Steven R Clark, Varun Soti

**Affiliations:** 1 Dermatology, Lake Erie College of Osteopathic Medicine, Elmira, USA; 2 Pharmacology and Therapeutics, Lake Erie College of Osteopathic Medicine, Elmira, USA

**Keywords:** recurrence, efficacy, carbon dioxide laser, surgical deroofing, hidradenitis suppurativa

## Abstract

Hidradenitis suppurativa (HS) is a chronic, inflammatory skin condition that causes pain and discomfort in various body regions. This review explores the comparative effectiveness of two surgical techniques, namely, surgical deroofing and carbon dioxide laser therapy, in managing symptomatic HS, particularly in patients with Hurley stage I-III disease. We conducted a systematic literature search on PubMed and ClinicalTrials.gov. The clinical evidence suggests that surgical deroofing and carbon dioxide laser treatment are effective strategies for managing symptomatic HS. However, a comprehensive analysis of 1,120 patients indicates a higher recurrence rate with surgical deroofing. Further investigation into short-term and long-term follow-up data revealed comparable recurrence-free rates within 12 months post-procedure. Beyond 12 months, carbon dioxide laser treatment exhibited slightly higher recurrence-free rates, which necessitate more extensive studies for validation due to the limited sample size. In addition, surgical deroofing demonstrated quicker healing times, while carbon dioxide laser therapy showcased varying timelines, with primary closure after laser excision presenting a two-week healing time. Both procedures reported high patient satisfaction, emphasizing the need for personalized treatment decisions. Therefore, further research is essential to evaluate the efficacy of each treatment modality considering individual patient profiles and disease severity. It will benefit individuals affected by HS, leading to better health outcomes.

## Introduction and background

Hidradenitis suppurativa (HS), a chronic and debilitating inflammatory skin disorder, casts a considerable shadow on the lives of those afflicted. This malady, also known as acne inversa, manifests as painful nodules, abscesses, and tunnels under the skin, most frequently affecting the inguinal and perianal regions, axillae, and inframammary areas [1]. HS poses a substantial burden on patients, significantly impairing their quality of life due to persistent discomfort, pain, and the potential for disfigurement [2].

Despite its prevalence, HS remains a therapeutic challenge. The disorder is more prevalent in women, with a significantly higher occurrence in African-American populations than in White populations [3]. In recent US statistics, the estimated prevalence of HS is approximately 1% of the population [4]. This condition is often associated with obesity and tobacco use, further complicating its multifaceted etiology [5].

Recurrent painful nodules, abscesses, sinus tracts, and scarring characterize the clinical presentation of HS. These manifestations contribute to a significant decrease in the quality of life for individuals grappling with this condition. HS's chronic and recurrent nature underscores the importance of identifying effective treatment strategies [6].

In recent years, surgical interventions have emerged as promising avenues for managing symptomatic HS. Among these interventions, surgical deroofing and carbon dioxide laser therapy have gained attention for their efficacy in mitigating symptoms and improving patients' overall well-being. Surgical deroofing involves the removal of affected tissue and drainage of abscesses, while carbon dioxide laser therapy utilizes laser energy to target and ablate the diseased tissue [7].

Surgical deroofing has shown promise as a viable treatment option for HS lesions. The procedure aims to alleviate symptoms by addressing the underlying pathology, offering relief to patients grappling with the physical and emotional toll of HS. However, despite its efficacy, concerns linger regarding recurrence rates and the potential for complications [7].

On the other hand, carbon dioxide laser therapy has emerged as an innovative approach, harnessing the power of laser technology to target and remove affected tissue precisely. This technique has gained attention for reducing scarring and accelerating the healing process. The application of carbon dioxide laser therapy in HS treatment underscores the evolving landscape of dermatological interventions, emphasizing the need for a nuanced understanding of its comparative effectiveness [8].

This paper contributes to this understanding by comparing the effectiveness of surgical deroofing and carbon dioxide laser therapy in managing symptomatic HS lesions. By delving into existing literature, scrutinizing clinical evidence, and analyzing outcomes, this paper aims to comprehensively evaluate these surgical interventions, shedding light on their respective merits and limitations.

This review compares the effectiveness of surgical deroofing and carbon dioxide laser therapy in treating symptomatic HS lesions, Hurley stage I-III. By critically assessing available data and analyzing recurrence rates, healing times, and patient satisfaction, this paper seeks to offer insights into the relative merits of these interventions. Understanding the nuanced differences between surgical deroofing and carbon dioxide laser therapy is crucial for clinicians and healthcare providers in making informed decisions tailored to individual patient profiles and preferences. The synthesis of existing knowledge and the analysis of clinical outcomes offer a nuanced perspective that can inform evidence-based practices, ultimately enhancing the overall care and patient outcomes for individuals grappling with symptomatic HS lesions.

## Review

Methods

**Figure 1:**
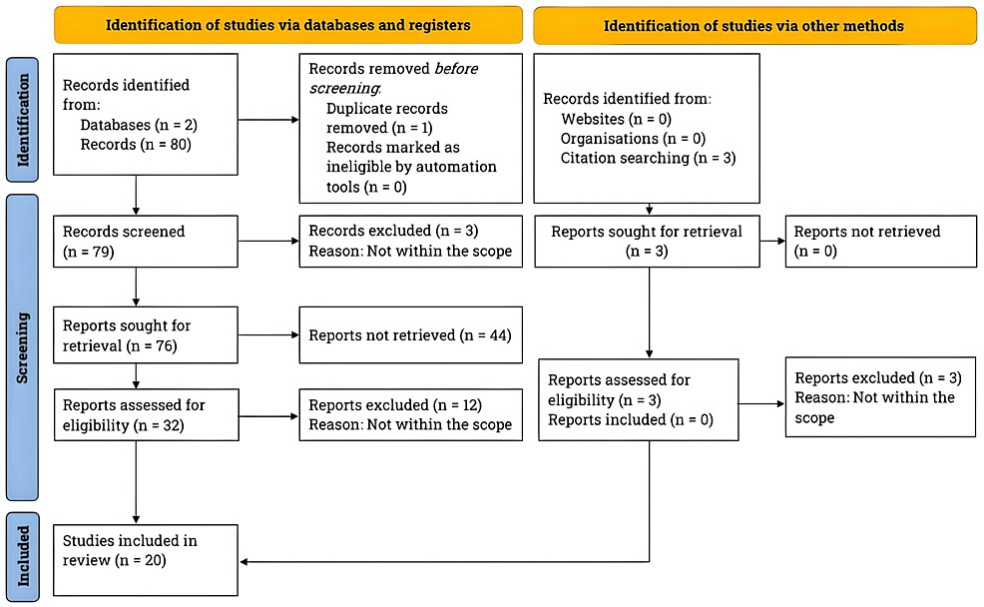
Research methodology: an overview of the PRISMA flowchart for literature search and study selection. We followed the PRISMA guidelines and conducted a comprehensive literature search on PubMed and ClinicalTrials.gov. Our exclusive focus was on studies published within the last five decades investigating the effectiveness of surgical deroofing and carbon dioxide laser interventions for treating hidradenitis suppurativa. *n*, number; *PRISMA*, Preferred Reporting Items for Systematic Reviews and Meta-Analyses

We carried out a literature search using PubMed and Clinicaltrials.gov following the Preferred Reporting Items for Systematic Reviews and Meta-analyses (PRISMA) guidelines between February 2023 and April 2023 (Figure 1) [9].

The search strings “hidradenitis suppurativa carbon dioxide laser” and “hidradenitis suppurativa deroofing” returned 36 and 38 results on PubMed, respectively. However, the exact search on ClinicalTrials.gov returned only two and four results, respectively. We meticulously screened and included only relevant studies in this review (see Table 1 for the study selection criteria).

**Table 1:** Criteria for study selection: a tabular representation. We included relevant studies on the effectiveness of surgical deroofing and carbon dioxide laser techniques in treating hidradenitis suppurativa. We only considered studies published in English from 1960 to 2023 that met the inclusion criteria outlined in this table.

Inclusion criteria	Exclusion criteria
Randomized controlled trials	Preclinical studies
Non-randomized controlled trials	Systematic reviews
Prospective clinical studies	Meta-analyses
Observational studies	Narrative reviews
Pilot studies	Commentaries
Retrospective studies	Opinions
Case series	
Case reports	

Understanding the diagnosis and staging of HS

HS is a long-term inflammatory skin condition that can lead to significant discomfort and distress for patients. However, diagnosing HS can be challenging, as it is typically based on clinical symptoms rather than histological examination. It means that the condition is often only identified once a patient presents with typical, recurring HS lesions, including nodules, fistulas, and abscesses in areas of the body where HS is known to occur. Unfortunately, this delay in diagnosis often means that patients have a more advanced presentation, which significantly delays proper diagnosis and treatment [10].

A tool commonly used to classify HS is Hurley staging. Introduced in 1989, it remains valuable for identifying the extent of the disease and planning appropriate management strategies. HS is typically classified as Stage I, II, or III. Stage I disease is the mildest form, characterized by chronic or recurrent lesions in typical locations, while Stage II involves scarring in one area and a small number of fistulae. Stage III disease is the most severe, with extensive fistulae and scarring. However, it is crucial to note that most patients have Stage I or II disease and never advance to Stage III. Understanding the various stages of HS can help patients and providers approach management and treatment with greater confidence and clarity [11].

Standard treatments for HS

Standard treatment protocols are based on the severity of the disease and include lifestyle modifications, pharmacologic therapy, and surgical management. Typical first-line pharmacologic management includes topical antiseptics, such as chlorhexidine and the topical antibiotic clindamycin 1% for mild Hurley Stage I and II disease. First-line systemic antibiotics are typically oral tetracyclines, doxycycline 100 to 200 milligrams (mg) daily for 12 weeks. Oral systemic therapies are commonly used for mild-moderate disease. Moreover, for severe forms, a combination of rifampin 600 mg daily and clindamycin 300 mg twice daily for 12 weeks is indicated. Immunotherapeutic drugs can be considered after the failure of antibiotics. Some of these immunotherapeutics include adalimumab and secukinumab. HS surgical management is reserved for those with a disease without any clinical response to various nonsurgical therapies. The standard options for surgical management include deroofing, wide surgical excisions, neodymium:yttrium-aluminum-garnet (Nd:YAG) laser therapy, carbon dioxide laser therapy, and then incision and drainage of lesions [12].

Assessing the treatment effectiveness and quality of life of HS patients

When gauging the effectiveness of treatment for HS, measuring the recurrence rate of lesions takes top billing. This model is a must to diagnose HS and prevent future occurrences. Another commonly used model is the hidradenitis suppurativa clinical response (HiSCR). This model has three specific criteria to evaluate whether a patient responds to treatment: no increase in fistulae, no increase in abscesses, and at least a 50% reduction in active inflammatory nodules or abscesses [13]. 

Other models used to assess treatment effectiveness include the modified Sartorius score, which evaluates the number and positioning of lesions, and the HS Physician’s Global Assessment, a simple way to measure disease severity that is not as specific as the HiSCR [14]. In addition, Dermatology Life Quality Index (DLQI) scores are widely used to assess the effects of HS on the overall quality of life [15].

The DLQI score is a standardized questionnaire comprising 10 questions. Each question carries a point value, and the total score indicates the severity of disease effects on the quality of patient life. This score has been used since the 20th century and is still effective for many skin conditions. Hence, these models work best for the most comprehensive assessment of HS treatment success [15].

Managing HS with surgical deroofing

Surgical deroofing is widely employed in the mild-to-moderate surgical management of HS, besides applying laser therapy and local excision to HS lesions. Since its introduction in 1959, the process has evolved to become more effective by exposing the floor of the lesion [16]. The process involves several steps, including removing the roof of the entire lesion, exposing the floor of the underlying sinus tract, and cleaning any purulent material that may be present. 

The initial incision is typically made by cold steel or electrosurgical dissection using a wire loop tip of the electrosurgical generator or a scalpel/scissors, followed by blunt-probing each lesion to check for potential branching tracts from the original visible lesion; any fistulae are subsequently deroofed, leaving the floor of the lesion exposed. Lesions are then debrided to remove purulent or adherent material. The resulting deroofed wounds are left to heal by secondary intention, with minimal scarring [16].

van der Zee et al. (2010) conducted a study to evaluate surgical deroofing’s effectiveness in managing HS in a cohort of 44 Hurley Stage I and II patients. The study also aimed to assess patient satisfaction with the procedure. The research team examined 88 HS lesions in 44 patients, all treated by a single clinician. The study cohort comprised 41 females and 3 males, with a mean age of 35 at the time of the procedure and a median body mass index (BMI) of 26.8. Most of the lesions subjected to deroofing were located in the groin and axilla, with some occurring in the buttocks [17]. 

The efficacy of the surgical deroofing procedure was evaluated based on the recurrence rate and overall patient satisfaction, rated on a scale of 1-10, with 10 signifying maximum satisfaction. The researchers found that 83% of patients showed no disease recurrence after a median follow-up of 34 months. Only 15% experienced a recurrence, which presented as an inflammatory nodule characterized by a size of less than 0.5 centimeters (cm) from the adjacent scar. Furthermore, the median patient satisfaction score was eight, and 90% of patients indicated they would recommend the procedure to other HS patients. Although the study had some limitations, such as the absence of a control group and statistical analysis, its findings demonstrated the efficacy of deroofing in preventing HS recurrence in chronic patients at Hurley Stages I and II. In addition, it showed that deroofing was a cost-effective procedure that provided fast and easy relief of symptoms, with surgeons being able to perform it on the same day of diagnosis [17]. 

A retrospective study conducted by Kohorst et al. (2016) aimed to assess the recurrence rates in 590 patients who had undergone surgical management for HS between 1976 and 2012. The study focused on the effectiveness of three surgical procedures, i.e., wide excision, surgical deroofing, and incision and drainage. The study cohort consisted of predominantly white (91%) and male (57.4%) patients, including smokers. The study protocol followed up on the patients from one to 6,691 days post-procedure. Recurrence was a newly described disease adjacent to or within the previously operated area [18].

The Kaplan-Meier method determined that over 75% of patients had no recurrence. However, complications were reported in 15 of the 590 patients within 30 days of the procedure, of which nine were cellulitis. The other complications included two skin graft losses, one each for wound dehiscence, neuropathic pain, retained foreign body, and hematoma. The study revealed that incision and drainage had the highest recurrence rate, whereas wide excision and deroofing had a significantly lower recurrence rate. However, there was no significant difference in the recurrence rate between wide excision and deroofing (hazard ratio or HR 1.0). The findings demonstrated that younger patients and patients with operations at multiple locations had a higher recurrence rate than older patients or those with only one surgical site, with an HR of 0.8 and 1.6, respectively [18]. 

Moreover, the study showed that deroofing was superior to wide excision, with the latter posing no benefit in preventing HS recurrence. However, the researchers did not report specific recurrence rates for each procedure, a significant study limitation. Notably, the patient cohort did not represent the groups most at risk of refractory cases of HS. Nonetheless, the researchers directly compared surgical groups to determine their effectiveness in treating HS recurrence and used statistical analyses to validate their findings [18].

A recent retrospective cohort study by Ravi et al. (2022) aimed to evaluate the efficacy of deroofing compared to wide excision in patients diagnosed with HS. The study was conducted at the Department of Dermatology, University of North Carolina Chapel Hill, and involved 78 HS patients undergoing 194 surgical procedures. The study aimed to determine the recurrence rates of HS in patients who underwent deroofing or wide excision procedures and the patient’s satisfaction with the procedures and post-procedure outcomes [19].

The study population had an average age of 35.1 years, and most patients (83%) were female. Obesity and cigarette smoking were observed as coexisting conditions in some patients. Out of the 194 total procedures, 129 were deroofing procedures. The study’s results showed that over 67% of patients who underwent deroofing procedures had no recurrence of HS. On the other hand, excision with or without closure had a higher recurrence rate (p = 0.04). Interestingly, patients over 35 had a significantly lower recurrence risk than those under 25 (p = 0.003) [19].

In addition, the study found that deroofing allowed for a faster return to regular activity and had a significantly shorter healing time than wide excision (p = 0.02). Post-procedure satisfaction surveys showed that 87% of patients who underwent deroofing were satisfied with the procedure, and 86% were satisfied with the outcome. Nevertheless, the study acknowledged potential limitations such as recall bias due to some patients struggling to remember their exact procedure and location. These findings further support the application of deroofing as a practical and preferred procedure, as reflected by its lower recurrence rate in patients who underwent the procedure compared to those who underwent wide excision [19].

Dahmen et al. (2019) conducted a prospective study to evaluate the efficacy of a modified deroofing procedure for treating HS. The study enrolled 52 Hurley Stage II/III patients with 96 HS lesions who underwent modified deroofing. During this procedure, surgeons removed the tough fibrotic tissue from the sinus floor or surrounding walls. No patients received systemic treatment preoperatively, and only topical therapy was administered. Recurrence was defined as the appearance of inflammatory nodules within a 2 cm radius of the scar following the procedure [20].

Of the 45 patients who were followed up for at least 18 months, 83% remained recurrence-free. Moreover, 86% of the treated lesions remained non-recurrent. The only complication observed was bleeding from the surgical site, which occurred in a small number of cases. Specifically, 89 out of the 96 lesions did not bleed. The time required for surgical wounds to heal was 5.2 weeks, significantly longer than the healing time observed in van der Zee et al.'s study (2010). This discrepancy may be due to the more extensive surgical wounds incurred during modified deroofing. However, it is essential to note that, as with van der Zee et al. (2010) [17], the lack of a control group and statistical analysis limits data interpretation and generalization of this study’s findings. Future studies could benefit from including a third group of patients undergoing standard deroofing, allowing for a more accurate comparison with modified deroofing. Nevertheless, the study’s results demonstrated that modified deroofing effectively achieved clinical response and prevented lesion recurrence in treated areas in patients with HS [20].

Blok et al.’s (2015) retrospective analysis evaluated the efficacy of two surgical techniques, deroofing and skin-tissue-sparing excision with electrosurgical peeling (STEEP), in treating HS under general anesthesia. STEEP, a method similar to wide excision, involves removing diseased tissue while preserving as much healthy tissue as possible. The study included 113 HS patients who underwent 482 operations between 1999 and 2013. The sample consisted mainly of females (68%), except patients with a BMI over 35, those under 18, mentally disabled patients, or deceased individuals. The great majority of patients had Hurley Stage II HS, while all had been diagnosed with Hurley Stages I to III [21].

The study’s median follow-up period was 43 months. Within that time, 84% of patients had no complications, with hypergranulation being the most common post-procedure complication. Notably, 76% of patients favored surgical treatment, and 70% did not experience disease recurrence. Gender was a crucial indicator of HS recurrence, with females being 2.85 times more vulnerable than males (p = 0.0369), without specifying the causing surgical procedure. The study also suggested that the deroofing method exhibited a comparable efficacy to the tissue-saving STEEP technique but was more cost-effective and less time-consuming, making it a promising intervention for treating HS patients [21].

In a study conducted by Saito-Sasaki et al. (2022), two cases were presented where patients underwent surgical deroofing with adalimumab as a postoperative prophylactic. The first case involved a 58-year-old male with refractory HS in the buttock and axilla, who underwent deroofing, followed by a 10-month adalimumab regimen. However, he did not show significant improvement upon follow-up. Therefore, a second deroofing was performed, and the patient received two weeks of cephalosporin before initiating adalimumab for four months. The patient remained recurrence-free for one year after treatment [22].

The second case involved a 58-year-old male with disseminated HS throughout the buttock and scrotum, 15 years post-excision. After a complete assessment, the patient underwent deroofing followed by antibiotics and an adalimumab regimen. Remarkably, the patient received significant clinical benefits within two months. These findings suggest that surgical deroofing with adalimumab as a postoperative prophylactic may be an effective treatment approach for refractory HS. Future research studies with larger samples must confirm these results and identify the optimal treatment duration [22].

These cases demonstrate the limited effectiveness of deroofing as a standalone surgical option for recalcitrant or recurrent widespread lesions. Deroofing was effective in each case only after subsequent administration of antibiotics and adalimumab. Future research can build on these findings and assess the efficacy of adalimumab with and without deroofing in treating patients with recurrent HS [22].

Contrary to Saito-Sasaki et al. (2022) [22], Brown et al. (1986) documented four instances of refractory HS, effectively resolved with deroofing. Wide excision was not feasible in these cases since the HS was pervasive. The issues involved a 32-year-old man, a 36-year-old woman, and a 40-year-old man, while one of the patients, a 57-year-old man, expired due to unrelated cardiorespiratory issues after finishing his treatment but before publication [23].

Patients underwent multiple deroofing operations because of the extensive lesions, demonstrating notable improvements in disease severity in operated areas. However, the 40-year-old man was misdiagnosed with a fistula-in-ano, and this delay caused extensive delays in receiving the appropriate treatment and subsequent therapeutic benefits. Prompt HS diagnosis in its early stages reduces the likelihood of disease progression [23].

This instance underlines one of the unique challenges of this ailment. Since the diagnosis is clinical-based and the diagnostic criteria, including chronic or recurring lesions, delays, allowing progression to later stages can be detrimental, posing more severe challenges for directed approaches. Therefore, attaining the most prompt and accurate diagnosis is fundamental to maximizing deroofing effectiveness [23].

It is worth noting that the researchers did not follow up with patients for recurrence, and their symptom improvement reports lacked quantitative measures, resulting in a comprehensive analysis limitation. Despite the noted issues, this case series highlighted deroofing’s efficiency in treating recurrent, widespread HS lesions [23].

Lin et al. (2016) reported on a case study of a 35-year-old male diagnosed with Hurley Stage III HS for deroofing. The man had severe involvement in his right gluteal region; the only identifiable postoperative complication was tolerable pain. With wound healing within 46 days after the procedure, no recurrence was noted at a three-month follow-up. A potential drawback of this case study was the short follow-up timeframe for recurrence. Despite this, the study adeptly demonstrated the value of deroofing in treating more severe HS cases with Hurley Stage III (differing from prior studies where Hurley Stage I or II patients were studied) [24].

Schultheis et al. (2021) presented a case report of a 31-year-old male with Hurley Stage II HS lesions on his lower abdomen, thighs, buttocks, and groin. The patient had a remarkable history of prior use of antibiotics, comprehensive excision surgery, and adalimumab to treat his HS. Despite steroid use, lesions recurred upon discontinuation. The researchers initially employed ustekinumab (an antagonist of interleukin 12 and 23) with biweekly LAight® therapy to treat the chronic and recurrent HS, but it did not provide the desired relief. Therefore, they implemented deroofing in two steps to treat all the affected areas. The wound healed without complications, and the patient reported significant improvement in his quality of life, as evidenced by his DLQI score of 14 and the numerical rating scale (for pain) score of 0. Two years later, he reported minor lesions recurrence in the treated areas, but LAight® therapy successfully resolved it without the need for deroofing again [25].

The researchers demonstrated the versatility of deroofing as an effective supplement to other treatment modalities, such as LAight® therapy, in treating chronic, recurrent, and severe HS. They also highlighted the deroofing technique’s potential to enhance HS patients’ quality of life. These case findings suggested that deroofing can be an effective treatment option, particularly when refractory to other treatments [25].

Haoxiang et al. (2013) published a case series that featured eight cases where patients underwent a deroofing procedure utilizing “modified abscess drainage.” This approach involved the removal of limited sinus tract roofs while exploring with a probe and piercing through openings in the sinus when there was communication between tracts and leaving them open to heal over time. Most patients were HS Hurley Stage II or III and males, with one having diabetes. All patients received a combination of intravenous levofloxacin, azithromycin, and oral metronidazole for five to seven days, both before and after the procedure. The researchers followed up on patients three months post-operation [26].

The surgeons operated on 28 lesion sites in eight patients, resulting in 82.1% of patients showing either a complete recovery or a significant improvement of symptoms. Postoperative complications included pain, fever, and bleeding, with annoyance present in 50% of patients. Patients did not report recurrence at the three-month follow-up. Of note, surgeons did not remove the lesion roof of several lesions with communicating sinuses, leaving them to heal by allowing them to drain. This modified approach showed clinical benefits, reinforcing deroofing’s utility, its applicability with different techniques, and its significant advantage in the healing process [26].

HS can be a harrowing and debilitating disease that is hard to treat. Surgical deroofing has been a successful treatment option since 1959 and has proven to be versatile, cost-effective, and more accessible than routine radical excision [10,17]. However, more studies must be done to properly randomize and compare deroofing against other surgical treatments. HS has many treatment options; each patient’s best option might differ. One of these options is the use of laser therapy for the treatment of lesions. A couple of lasers are in use for treating HS, but the carbon dioxide laser is the most widely used. Please refer to Table 2 for a brief summary of the critical studies and their findings.

**Table 2:** Surgical deroofing: key studies showing the effectiveness in treating HS patients. The table presents a compilation of critical studies demonstrating the effectiveness of deroofing as a treatment for HS patients. It showcases the summary of essential study designs and findings relating to the successful resolution of HS lesions, recurrence-free time, and recurrence. *%*, percentage; *HS*, hidradenitis suppurativa

Authors	Type of study	Sample size	HS staging	Findings
Van der Zee et al. (2010) [17]	Prospective clinical	44 patients (88 HS lesions)	Hurley Stages I and II	Of the treated lesions, 82.95% were successfully resolved and showed no recurrence at a median follow-up of 34 months.
Kohorst et al. (2016) [18]	Retrospective cohort	590 patients	Hurley Stage III (81%); Hurley Stages I-II	Out of all the patients treated, 75% showed successful resolution of HS lesions with no recurrence during their post-procedural follow-ups. The length of follow-up varied from one day to as long as 6,691 days after the procedure.
Ravi et al. (2022) [19]	Retrospective cohort	78 patients (194 procedures)	Hurley Stages II and III	Of all the surgical deroofing procedures performed, 67% were successful, with no recurrence in an average of 79 days during post-procedural follow-up.
Dahmen et al. (2019) [20]	Prospective clinical	52 patients (96 HS lesions)	Hurley Stages II and III	Of the treated lesions, 86% were successfully resolved and showed no recurrence upon an 18-month follow-up.
Blok et al. (2015) [21]	Retrospective cohort	113 patients (482 procedures)	Hurley Stages I to III	Of all the surgical deroofing procedures performed, 70% were successful, with no recurrence at the post-procedural follow-up, which spanned a median of 43 months.
Saito-Sasaki et al. (2022) [22]	Case report	2 patients	Not reported	The first patient remained recurrence-free for one year after treatment. The second patient received significant clinical benefits within two months.
Brown et al. (1986) [23]	Case series	4 patients	Not reported	All patients with refractory HS lesions had successful resolution. One patient was initially misdiagnosed, which delayed the treatment but eventually had a successful resolution of HS lesions. There was no follow-up with patients for recurrence.
Lin et al. (2016) [24]	Case report	1 patient	Hurley Stage III	The patient had a successful resolution of HS lesions. No recurrence was noted at a three-month follow-up.
Schultheis et al. (2021) [25]	Case report	1 patient	Hurley Stage II	Post-procedure, HS lesions were successfully resolved, and the patient remained recurrence-free for up to two years. Minor lesions recurred in the treated areas, but re-deroofing was not required; the LAight® therapy successfully resolved them.
Haoxiang et al. (2013) [26]	Case series	8 patients (28 HS lesions)	Hurley Stages II and III	Of all patients treated, 82.1% either wholly recovered or significantly improved. No recurrence was noted at the three-month follow-up.

Using carbon dioxide laser in HS treatment

Next, this review focuses on the second set of surgical techniques, namely, carbon dioxide laser, which can be used for various purposes, such as vaporization, marsupialization, and excision of HS lesions. Vaporization involves the successive evaporation of tissue layers with the laser until healthy tissues are exposed and all the diseased and fibrotic tissues have been removed. On the other hand, the excision method involves removing all the diseased tissue with healthy margins around the lesion, using a carbon dioxide laser to establish better hemostasis and more efficient procedures. Lastly, marsupialization occurs when the base of the previously excised lesion is vaporized with a carbon dioxide laser, resulting in a pocket of healthy tissue named marsupialization. Carbon dioxide laser is a versatile surgical technique that can manage HS lesions with varying degrees of success [8].

Lapins et al. (1994) conducted a study on 24 patients, 21 of whom were women and three were men. Each of these patients had experienced at least three episodes of recurrence in the year before the study treatment, and all were Hurley stage II at the time of surgery. Patients with other diseases prone to the formation of fistulas, such as Crohn’s disease and ulcerative colitis, were excluded from the study. Some coexisting conditions included type 1 diabetes, hypothyroidism, and obesity [27].

The study involved selecting the most symptomatic lesions and then ablating them using a 30-watt carbon dioxide laser, per the protocol. Of the 24 patients treated, 22 did not experience any recurrence within 5 cm of the treated area. Four patients had lesions in the same region, but not the treated area, and 10 had lesions in a different anatomical region. This suggests that these lesions resulted from natural disease processes rather than recurrence post-ablation [27].

During the follow-up period, eight patients did not develop any new lesions, with a total mean follow-up time of 27 months after carbon dioxide ablation. The average time to heal was four weeks. The study concluded that carbon dioxide ablation effectively prevented most patients’ recurrence of symptomatic lesions [27]. 

Notably, the study was conducted without a control group or statistical analysis of results. However, the generated data indicate that the carbon dioxide laser could effectively prevent the recurrence of HS lesions, with 91.67% of the treated lesions showing no signs of a relapse. Laser therapy, much like deroofing, is an established modality for treating symptomatic lesions [27].

Mikkelsen et al. (2015) conducted a retrospective study to analyze the recurrence rates and overall patient satisfaction following carbon dioxide laser treatment by vaporization and healing by secondary intention. The study comprised 58 patients, out of which 58 were interviewed. The study participants were divided into two groups based on their age, above and below 45 years, and the results were obtained through phone or in-person interviews. The study participants had coexisting conditions, such as obesity and smoking, and the gender distribution was 40 women and 10 men [28].

The study results indicate that 70% of the patients did not report any recurrence within the borders of the treated lesions, and 95% of the patients registered at least a slight improvement in the status of their HS. Furthermore, 91% of the treated patients recommended carbon dioxide laser treatment to others. The follow-up of the patients was conducted at an average of 25.7 months after the treatment. However, one of the study’s restrictions is that the results were obtained through patient-reported data, with some patients being interviewed over the phone, which introduces recall bias into the study [28]. Despite this limitation, the study shows similar patient satisfaction rates to that of deroofing and similar recurrence rates to the deroofing study conducted by Ravi et al. (2022), indicating the usefulness of laser therapy [19,28].

Lapins et al. (2002) conducted a retrospective study on carbon dioxide laser surgery to evaluate the effectiveness of a scanner-assisted surgical technique. The study aimed to analyze this method’s efficacy in treating Hurley stage II HS patients who had at least three recurring lesions in the past year. The study included 31 females and four males with previous pharmacologic and surgical interventions, including carbon dioxide laser surgery [29].

The surgical technique employed optomechanical scanners attached to the carbon dioxide laser, which assisted in the ablation of diseased tissue by increasing precision and reducing laser exposure time. The method involved the creation of a spiral beam that ablates successive tissue layers until yellow fat is exposed, followed by healing by secondary intention [29].

The results of the study were obtained through a questionnaire. The average healing time was four weeks, with 88.2% of patients experiencing no recurrence of the treated lesion within six months. Only one patient reported that their condition had worsened, while 31 of the remaining 33 indicated that they had experienced symptomatic improvement in their HS. Only one surgical scar became hypertrophic, which was treated successfully. However, one limitation of this study is that the results were obtained through a patient questionnaire, which may introduce recall bias into the analysis [29].

The authors sought to study the vaporization technique, similar to their 1994 study [18], but with slight modifications to improve lesion ablation. The study yielded a similar ability to prevent the recurrence of lesions at 88.2% compared to 91.67% recurrence prevention. Over 90% of patients reported improvement in their symptoms, demonstrating that carbon dioxide laser therapy directly benefits patients and improves their symptoms [29].

Hazen and Hazen (2010) described using carbon dioxide laser excision and marsupialization to treat sinus tract patients. In this study, 61 patients were selected, of which 42 were women. The procedure involved excising the diseased tissue using a carbon dioxide laser and vaporizing the base of the surgical area and margins using the same laser to account for the marsupialization component. The study involved 154 procedures; only two patients experienced a recurrence within the treatment area. The disease recurrence in treated areas was found to be 4.1 years on average throughout follow-up. Postoperative complications included hypertrophic granulation tissue, cellulitis, sweets syndrome, and wound dehiscence. These complications were successfully treated. The average time for secondary intention healing was found to be 8.8 weeks, which is longer than some other secondary healing procedures due to the extensive nature of the procedure [30].

A constraint of this study is the varied follow-up time range of one to 19 years, which makes it challenging to analyze factors that influence recurrence. In addition, there were few African American patients, and statistical significance could not be confirmed on whether this played a role in the number of treatments required. However, the reported data in this study show favorable outcomes in terms of post-surgical recurrence rate, with only a 3.28% recurrence rate, which is the lowest among all the remedies proposed thus far. The study findings demonstrate that carbon dioxide laser excision and marsupialization are promising therapeutic choices for recurrence prevention. Although further comparative studies are needed, this study’s results are promising. Obese patients require more treatments than non-obese patients, averaging over seven treatments, while non-obese patients require less than three treatments. Obesity is significantly associated with more treatments required (p = 0.01) [30].

The study conducted by Azim et al. (2018) focused on combination laser therapy for Hurley Stage I or II HS patients. The cohort consisted of 30 adult HS patients not diagnosed with systemic disease or who had prior treatment for HS within two weeks of the study. Out of the initial pool, only 20 patients (11 men, nine women) met the inclusion criteria and were randomized. It is worth noting that 14 patients had a BMI above average, and nine patients had undergone previous surgical treatment for HS [31].

Each patient received two treatments for each side of their body, as most patients had bilateral symmetric symptomatic HS lesions. One side received only long-pulsed Nd:YAG laser therapy to destroy hair follicles, while the other received fractional carbon dioxide laser therapy followed by long-pulsed Nd:YAG laser therapy. Four treatments were administered, with two weeks between each treatment [31]. 

The results showed that the carbon dioxide laser therapy, combined with the Nd:YAG long-pulsed laser, significantly reduced the recurrence rate. In comparison, only 20% of the patients who received the long-pulsed Nd:YAG laser therapy reported no recurrence. Meanwhile, 55% of the patients who received carbon dioxide laser therapy in conjunction with Nd:YAG long-pulsed laser therapy experienced no recurrence after three months. The difference was statistically significant (p = 0.048) [31]. 

It is worth noting that this study has a limitation as it does not control for a side receiving only carbon dioxide laser therapy. However, the data demonstrate the effectiveness of the vaporization technique using the carbon dioxide laser in supplementing the Nd:YAG laser therapy. Although 45% of the patients experienced a recurrence, recurrence was defined as the activation of a presently treated lesion or the appearance of a new one. Carbon dioxide laser therapy has proven successful in treating and preventing the recurrence of symptomatic lesions. Nonetheless, there is a need for further studies to determine its effectiveness in preventing lesions over uninvolved areas [31]. 

Finley and Ratz (1996) reported a case series of seven patients treated with carbon dioxide laser excision. All the patients were women aged between 20 and 46 years and had received prior treatments for their lesions before the laser treatment. The lesions were excised with 3-4 millimeter margins and allowed to heal by secondary intention. The healing period ranged from four to 11 weeks, with most patients recovering at the six-to-seven-week mark. The postoperative period ranged from 10 to 27 months; only one recurrence was noted. One patient experienced temporary paresthesia and a stricture, one suffered from a candidiasis infection, which was successfully treated, and one required manual debridement of the surgical wound [32]. 

One of the study’s drawbacks is its small sample size, which is attributed to the fact that it is a case series. Another one is the unavailability of the exact ages of the participants, as they were only reported as a range. Patient age is a crucial factor needed to understand each case since advanced age is known to decrease the likelihood of recurrence of HS. The recurrence data reported in these cases are significant, with only one patient experiencing a lesion recurrence over two years. Even without statistical analysis, the fact that six patients had no symptomatic lesions in the area represents a substantial improvement in their quality of life following treatment [32].

Dalrymple and Monaghan (1987) conducted an empirical study using carbon dioxide laser treatment, which targeted the healing of HS. The study, which was retrospective in nature, involved six cases, five of which were females and one was male. Two other patients were undergoing treatment at the time of publishing. The age range of the patients was 20-43 years, with the youngest being male. Only one of the patients had never received treatment for HS. The tissue vaporization technique used the carbon dioxide laser, and patients underwent between one and four sessions. The wounds were left to undergo granulation and healed within three to seven weeks. Follow-up reviews were conducted at nine months and three years, where no recurrence was observed in any of the treated areas. Patients reported high satisfaction levels compared to the disease process, indicating a positive outcome of the surgery [33].

However, the study is limited because the patients’ ages were reported as a range. As explained, patient age plays a critical role in recurrence rates and is thus essential in understanding each case comprehensively. Despite the unavailability of exact ages, the vaporization technique had a positive outcome, with no recurrence observed in patients for at least three years. Moreover, the healing time was relatively similar to that of surgical deroofing, which further supports the efficacy of the technique in the HS treatment [33].

Madan et al. (2008) documented nine cases where refractory HS was treated using carbon dioxide laser surgery. The researchers selected the cutting mode of the laser and employed excision of diseased tissue in preference to vaporization for larger lesions. By contrast, more minor, singular diseased apocrine glands were vaporized. The wounds were closed after the operation rather than left to heal by secondary intention. The study included nine patients between 27 and 52 years of age, one of whom was male. A total of 27 lesions were treated, and the average time required for surgical wounds to heal was two weeks. Eight of the nine patients reported no recurrences in the treatment site, while only two reported active lesions adjacent to the site. Six patients noted no active lesions 12 months post-surgical intervention. Eight patients indicated in the questionnaire that they would recommend the procedure to others, demonstrating high patient satisfaction [34].

However, the study had a relatively smaller sample size and only one year of follow-up time. The study showed a much shorter healing time than earlier laser and deroofing studies since the wounds were closed instead of being left open to heal. This did not compromise treatment effectiveness since 88.9% of patients experienced no recurrence, demonstrating an advantage of the laser technique in possibly providing quicker healing times. Nonetheless, further research is necessary to analyze the outcomes of primary versus secondary intention healing following carbon dioxide laser excision [34].

A case report by Natarajan et al. (2014) illustrated the efficacy of carbon dioxide lasers in treating HS. The report describes a 34-year-old woman with HS that had spread from the left axilla to the groin, perineal, and right axillary areas. The team performed an excision over the left axilla with the carbon dioxide laser in continuous mode, followed by a split skin thickness graft to ensure proper healing and granulation tissue formation. One year after surgical treatment, the patient presented to the clinic with recurring lesions in the right axilla, groin, and perineum. However, the area that had received the graft was noticeably absent, as there had been no recurrence. Although scarring was present from the graft, no complications were reported [35].

Since this finding was limited to one patient, it warrants further exploration. Nevertheless, the case shows carbon dioxide laser excision can effectively prevent HS recurrence. Other areas in the patient that were not treated with the carbon dioxide laser had recurrences and required further treatment on follow-up. These findings align with the conclusions presented in other laser treatment studies, indicating that most patients can expect to be recurrence-free for some time post-treatment. Future studies with larger sample sizes are needed to confirm these findings and to explore the potential of carbon dioxide laser excision as a treatment for HS [35].

Jain and Jain (2012) conducted a study on nine patients; four were diagnosed with HS. The study aimed to assess the therapeutic efficacy of carbon dioxide laser-assisted deroofing of the sinus tract along with long pulse Nd:YAG laser treatment. The other five patients were diagnosed with a pilonidal sinus, a cyst or abscess often found near any location with apocrine glands and usually containing skin and hair debris. The study was conducted on female patients aged between 30 and 40, and the follow-up time was not specified [36]. 

The study used Nd:YAG laser first to remove all hair from the affected area, and then, after 15 days of hair removal, carbon dioxide laser was used to evaporate the roof of the sinus tracts and the collection at the bottom of the sinus tracts. The surgical wounds were left to heal by the second intention. The Nd:YAG laser therapy was repeated four to five times during the postoperative period of two to three months. The study reported that no lesions recurred at the treated sites, which illustrates the potential efficacy of the treatment modality [36]. 

The study did not report the exact age of the patients, which limits the data analysis slightly, as age is a significant factor in the likelihood of recurrence. However, the study provides evidence that combined carbon dioxide laser and deroofing, supplemented with hair removal laser therapy, can be an effective approach to treating HS. The demonstrated efficacy of this technique warrants further investigation [36]. Please refer to Table 3 for a brief summary of the critical studies and their findings.

**Table 3:** Carbon dioxide laser therapy: key studies showing the effectiveness in treating HS patients. The table presents the compiled data from crucial research studies highlighting the effectiveness of carbon dioxide laser therapy in HS patients. It concisely outlines the critical aspects of the study design and results concerning the resolution of HS lesions, the duration of recurrence-free periods, and/or the recurrence rates. *%*, percentage; *HS*, hidradenitis suppurativa; *Nd:YAG*, neodymium:yttrium-aluminum-garnet

Authors	Type of study	Sample size	HS staging	Findings
Lapins et al. (1994) [27]	Prospective clinical	24 patients	Hurley Stages II	Of 24 patients, 22 were treated successfully without any recurrence of symptoms, indicating a recurrence-free rate of 91.67% at an average follow-up time of 27 months.
Mikkelsen et al. (2015) [28]	Retrospective cohort	58 patients	Not reported	Of the patients treated, 95% registered improvement in their HS status, 70% did not report any recurrence within the borders of the treated lesions, and 91% recommended carbon dioxide laser treatment to others an average of 25.7 months after the treatment.
Lapins et al. (2002) [29]	Retrospective cohort	35 patients	Hurley Stages II	Of the patients treated, 31 experienced symptomatic improvement in their HS. Only one surgical scar became hypertrophic, which was treated successfully. About 88.2% of treated patients reported no recurrence at the six-month follow-up.
Hazen and Hazen (2010) [30]	Retrospective cohort	61 patients (154 procedures)	Hurley Stage III (late stage)	Of the patients treated, 96.72% exhibited successful resolution and remained recurrence-free. Only two patients reported recurrence after an average of 4.1 years post-treatment.
Azim et al. (2018) [31]	Prospective clinical	30 patients	Hurley Stages I and II	Of the patients treated with a combination of Carbon dioxide laser and Nd: YAG long-pulsed laser, 55% demonstrated successful resolution and remained recurrence-free at the three-month follow-up.
Finley and Ratz (1996) [32]	Case series	7 patients	Not reported	Of the treated patients, 85.71% remained recurrence-free at the follow-ups, which ranged between 10 and 27 months.
Dalrymple and Monaghan (1987) [33]	Retrospective cohort	6 patients	Severe, but stage was not reported	There was no recurrence in any treated areas at the nine-month and three-year follow-ups.
Madan et al. (2008) [34]	Case series	9 patients	Severe, but stage was not reported	Eight out of nine patients showed no recurrence at the treatment site, while only two reported active lesions adjacent to the site at the one-year follow-up.
Natarajan et al. (2014) [35]	Case report	1 patient	Not reported	There was no lesion reappearance at the treated site at the one-year follow-up.
Jain and Jain (2012) [36]	Case series	9 patients	Not reported	No lesions recurred at the treated sites at the follow-up. However, the follow-up time was not specified.

Discussion

**Figure 2:**
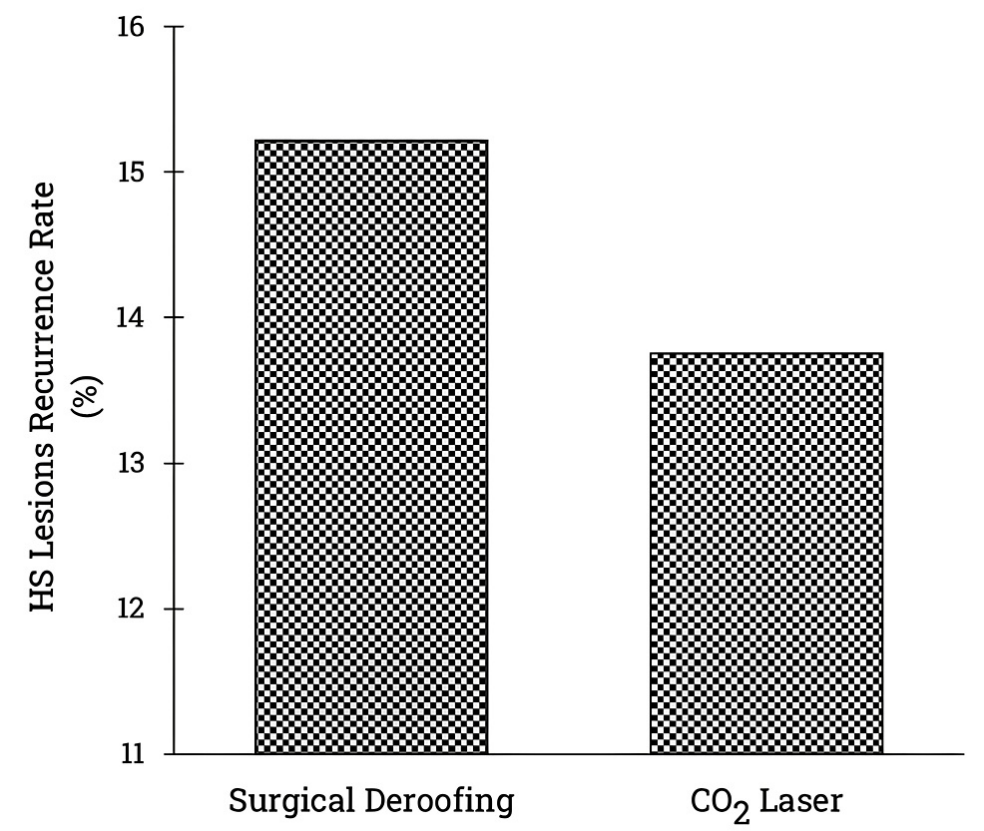
HS lesions recurrence rates for surgical deroofing and carbon dioxide laser techniques. This figure compares the recurrence rates of HS lesions between surgical deroofing and carbon dioxide laser. The x-axis indicates the two types of procedures (surgical deroofing and carbon dioxide laser), while the y-axis shows the recurrence rates. The data suggest that surgical deroofing has a higher recurrence rate than the carbon dioxide laser technique. Note: The data are aggregated from 18 studies, emphasizing the efficacy differences between the two treatment techniques for HS lesions. We excluded the studies conducted by Brown et al. [23] and Jain and Jain [36] due to the absence of patient follow-ups in their published reports. *%*, percentage; *HS*, hidradenitis suppurativa

The clinical evidence underscores the effectiveness of surgical deroofing and carbon dioxide laser treatment as viable approaches for managing symptomatic HS. Although their efficacy appears comparable, an analysis of 18 studies involving 1,120 patients indicates that surgical deroofing carries a higher recurrence rate compared to the carbon dioxide laser technique [17-22,24-35]. The visual representation in Figure 2 delineates the recurrence rates of HS lesions post-surgical deroofing and carbon dioxide laser procedures. Notably, data from Brown et al. (1986) and Jain et al. (2012) were excluded due to the absence of patient follow-up and reported recurrence rates [23,36].

**Figure 3:**
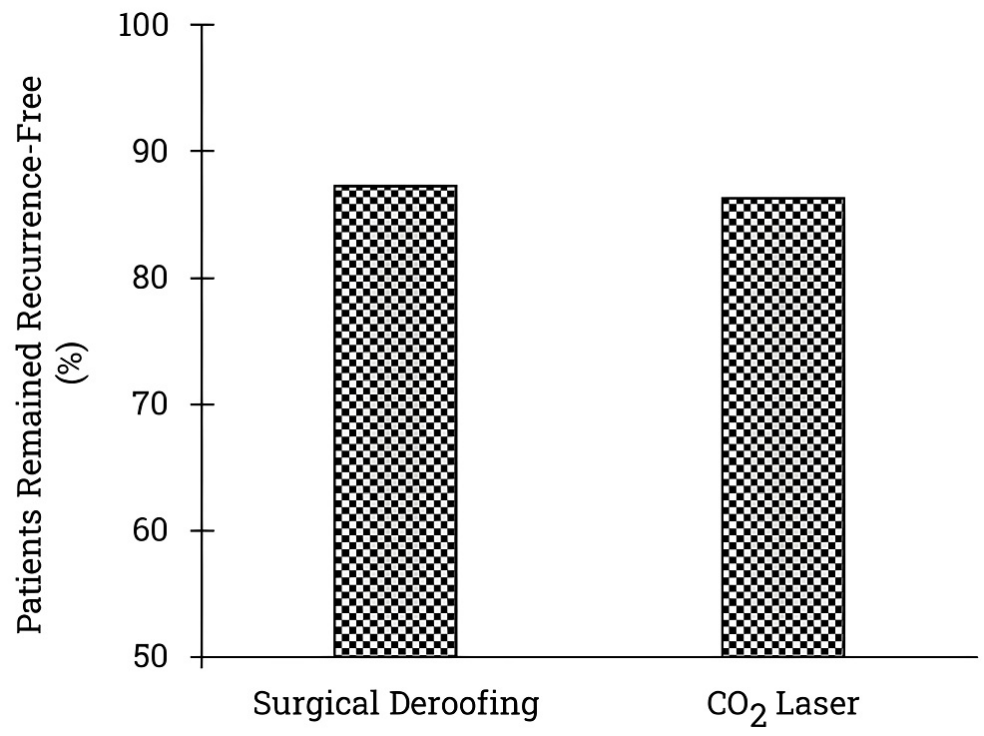
Recurrence-free HS patients after surgical deroofing and carbon dioxide laser treatment: follow-up at 12 months or less. The graph depicts the number of patients who remained recurrence-free after undergoing surgical deroofing or carbon dioxide laser treatment, with a follow-up period of up to 12 months. The data have been drawn from 10 studies with 89 patients undergoing surgical deroofing and 88 patients opting for carbon dioxide laser treatment. The x-axis illustrates the two procedures (surgical deroofing and carbon dioxide laser), while the y-axis represents the recurrence-free patients. Based on the statistical analysis, there is no significant difference between surgical deroofing and carbon dioxide laser treatment regarding the number of patients who remained recurrence-free for up to 12 months after the procedure. *%*, percentage; *HS*, hidradenitis suppurativa

We analyzed short-term and long-term follow-up data for patients undergoing surgical deroofing or carbon dioxide laser treatment to delve into recurrence-free survival rates. Segregating studies into two follow-up timeframes, namely, up to 12 months and beyond 12 months, we found no statistically significant differences in recurrence-free patients within the initial 12 months post-procedure based on aggregated data from ten studies involving 89 patients [19, 22, 24, 26-27, 31-36]. Figure 3 illustrates the recurrence-free rates up to 12 months following surgical deroofing and carbon dioxide laser treatment.

**Figure 4:**
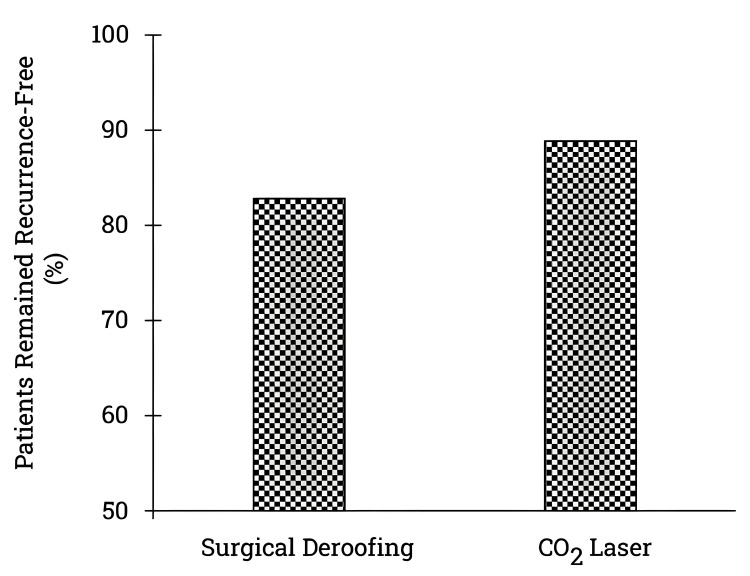
Recurrence-free HS patients after surgical deroofing and carbon dioxide laser treatment: follow-up beyond 12 months. The graph depicts the number of patients who remained recurrence-free after undergoing surgical deroofing or carbon dioxide laser treatment with a follow-up that exceeded 12 months. The data collated from 10 studies revealed that 800 patients underwent surgical deroofing, while 156 patients underwent carbon dioxide laser treatment. The x-axis illustrates the two procedures, surgical deroofing and carbon dioxide laser, while the y-axis exhibits the recurrence-free patients. Based on the inferential data analysis, the carbon dioxide laser treatment resulted in a slightly higher number of recurrence-free patients than surgical deroofing when followed up beyond 12 months after the procedure. *%*, percentage; *HS*, hidradenitis suppurativa

Inferential data analysis from 10 studies encompassing 956 patients indicated slightly higher recurrence-free rates beyond 12 months with carbon dioxide laser treatment than surgical deroofing. Notably, only 156 patients underwent carbon dioxide laser treatment, necessitating more extensive studies with larger sample sizes to validate superior recurrence-free rates beyond 12 months [17-18,20-21,25,27-28,30,32-33]. Refer to Figure 4 for the percentage of patients remaining recurrence-free beyond 12 months post-surgical deroofing and carbon dioxide treatment.

Transitioning to the investigation by Ravi et al. (2022), their findings accentuated the significantly abbreviated healing time and quicker return to normal activities associated with deroofing compared to wide excision, regardless of closure method [19]. By contrast, Dahmen et al. (2019) and Lin et al. (2016) reported a healing duration exceeding one month for surgical deroofing, implying multifactorial influences on the healing process [20]. While Ravi et al. (2022) identified disparities in healing times between non-Caucasian and Caucasian participants (p < 0.05), no significant correlation was established between smoking, obesity, and diabetes with healing times [19].

Distinct healing timelines emerged in carbon dioxide laser therapy, with excisional laser therapy displaying a minimum healing time of two weeks, typically falling within the three-to-eight-week range [32-34]. As per a singular study, primary closure following laser excision showed a two-week healing time, prompting further exploration into implications for recurrence or treatment efficacy [34]. On the other hand, deroofing demonstrated satisfaction values of at least 76% in three studies [17,19,21].

Patients undergoing carbon dioxide laser treatments reported high satisfaction levels, with up to 91% expressing willingness to recommend the procedure [28]. Despite similar 90% and 91% satisfaction rates for deroofing and carbon dioxide laser therapy, respectively, further research is vital to discern the optimal treatment based on individual patient profiles [17,28]. Notably, deroofing, conducted via electrosurgery, offers advantages in in-office execution and cost-effectiveness [17], distinguishing it from specific carbon dioxide laser techniques that may necessitate general anesthesia, potentially resulting in a longer, more complex, and more expensive procedure [35].

In-depth research is essential to assess the efficacy of each treatment modality across varying disease severity stages, providing insights into clinical responses and personalized treatment selection. Despite excision and marsupialization showing promise with a larger sample size and a 96.7% disease-free rate, complications, including Sweet's syndrome, were observed, highlighting the need for acknowledgment and consideration of associated risks with each procedure [30]. At present, complication rates are relatively low and indistinct between techniques, with complications like cellulitis showing similarity across approaches [18, 30].

Crucially, researchers in each study adopted varied definitions of recurrence, underscoring the necessity for standardized guidelines to facilitate accurate comparisons of surgical techniques in future investigations. In essence, each procedure demonstrates similar efficacy and entails associated risks, posing a challenge in determining optimal circumstances for application, necessitating further research to address diverse variables influencing intervention outcomes.

## Conclusions

This paper offers insights into the comparative effectiveness of surgical deroofing and carbon dioxide laser treatment in managing symptomatic HS. While both interventions have demonstrated efficacy in addressing symptoms, the data from various studies suggest a nuanced picture of recurrence rates and long-term outcomes. Recurrence rates for surgical deroofing tend to be higher than those for carbon dioxide laser treatment, highlighting the importance of considering the potential for disease recurrence when selecting an intervention. Moreover, longer-term follow-up data indicate that carbon dioxide laser treatment yields slightly higher recurrence-free rates, emphasizing the need for larger-scale studies to refine our understanding of long-term outcomes associated with these interventions. The heterogeneity in the definition of recurrence across studies underscores the need for standardized guidelines to facilitate meaningful comparisons in future investigations. Decision-making should factor in individualized patient profiles, disease severity, and procedural limitations. Further research is necessary to gain further insights and inform evidence-based clinical decisions that benefit individuals affected by HS.
